# Evaluation einer Basisschulung für Patienten mit rheumatoider Arthritis

**DOI:** 10.1007/s00393-020-00769-4

**Published:** 2020-03-16

**Authors:** C. Gerlich, I. Andreica, R. Küffner, D. Krause, H. J. Lakomek, A. Reusch, J. Braun

**Affiliations:** 1 Arbeitsbereich Medizinische Psychologie und Psychotherapie im Zentrum für psychische Gesundheit (ZEP), Universität Würzburg, Klinikstr. 3, 97070 Würzburg, Deutschland; 2 Rheumazentrum Ruhrgebiet, Ruhr-Universität Bochum, Herne, Deutschland; 3 Praxis Innere Medizin/Rheumatologie, Gladbeck, Deutschland; 4 Klinik für Rheumatologie und Geriatrie, Johannes Wesling Klinikum Minden, Minden, Deutschland; 5 Zentrum Patientenschulung und Gesundheitsförderung, Würzburg, Deutschland

**Keywords:** Rheumatoide Arthritis, Patientenschulung, Evaluation, Rheumatoid arthritis, Patient education, Evaluation

## Abstract

**Hintergrund:**

Ein neues Rahmenkonzept hat die flexible Ableitung und Nutzung von rheumatologischen Schulungsprogrammen für unterschiedliche Versorgungsbereiche ermöglicht. Auf dieser Grundlage wurde eine 5‑stündige Basisschulung für Patienten mit rheumatoider Arthritis (RA) entwickelt, es wurden rheumatologische Fachärzte und Psychologen trainiert, und dann wurde die Wirksamkeit nach dem Wirkmodell der Patientenschulung evaluiert.

**Methoden:**

Mit dem Studiendesign einer extern randomisierten Wartekontrollgruppenstudie mit 3 Messzeitpunkten wurde geprüft, wie sich die 5‑stündige Basisschulung auf das Erkrankungs- und Behandlungswissen sowie auf die Gesundheitskompetenz von RA-Patienten (*n* = 249) auswirkt. Weitere Fragen betrafen Einstellungsparameter, Kommunikationskompetenz, Erkrankungsauswirkungen und die Zufriedenheit mit der Schulung. Die Auswertungen erfolgten auf Intention-to-treat-Basis mit Kovarianzanalysen für die Hauptzielgrößen unter Berücksichtigung des Ausgangswertes.

**Ergebnisse:**

Die Analysen zeigen, dass die Basisschulung RA wirksam ist. Noch 3 Monate nach der Schulung verfügten die Schulungsteilnehmer über mehr Wissen und Gesundheitskompetenz als die Wartekontrollgruppe mit kleinem bis mittelgroßem Effekt (d = 0,37 bzw. 0,38). In den Nebenzielgrößen zeigten sich mit Ausnahme der Krankheitskommunikation keine weiteren Schulungseffekte.

**Diskussion:**

Die Basisschulung bietet eine gute Grundlage, auf der weitere Interventionen zur Verbesserung von Einstellungs- und Erkrankungsparametern aufbauen können. Sie eignet sich damit als zentraler Baustein für die rheumatologische Versorgung auf verschiedenen Ebenen.

**Zusatzmaterial online:**

Die Online-Version dieses Beitrags (10.1007/s00393-020-00769-4) enthält weitere Tabellen. Beitrag und Zusatzmaterial stehen Ihnen auf www.springermedizin.de zur Verfügung. Bitte geben Sie dort den Beitragstitel in die Suche ein, das Zusatzmaterial finden Sie beim Beitrag unter „Ergänzende Inhalte“.

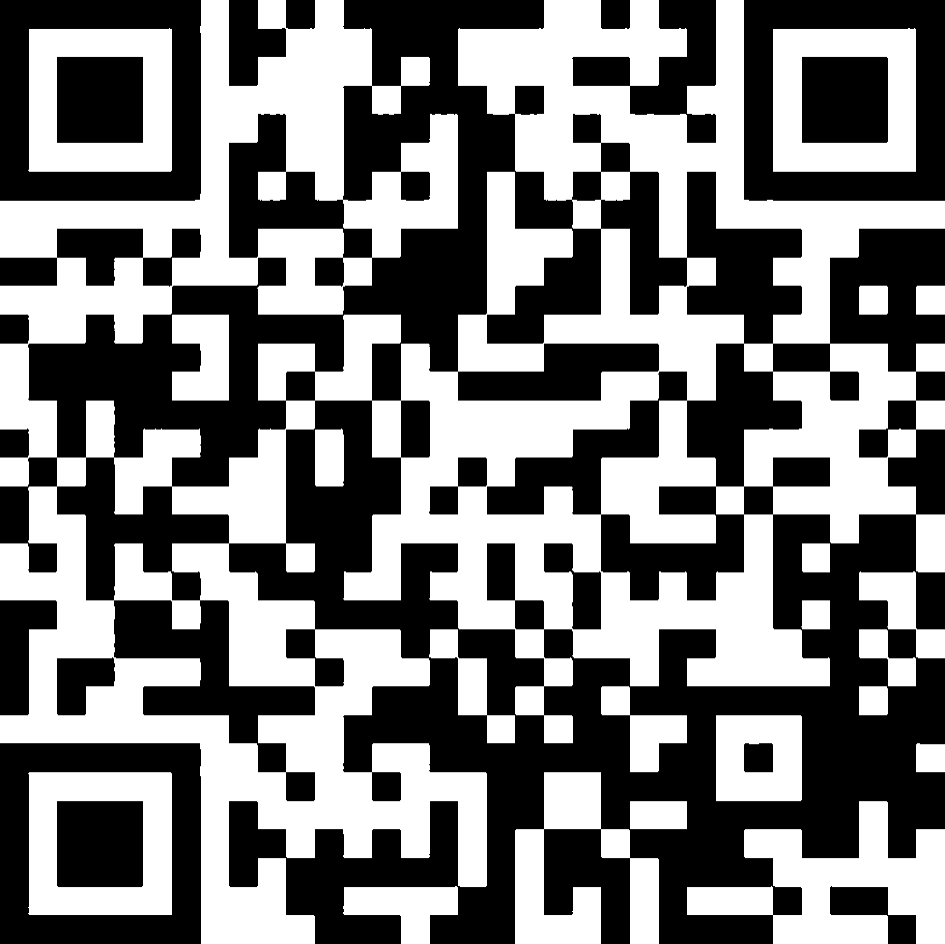

## Abstract (en)

**Background:**

A new conceptual framework has enabled the flexible development of rheumatological patient educational programs for different healthcare settings. On this basis, a 5‑h basic training program for patients with rheumatoid arthritis (RA) was developed to be used in specialized centers. Rheumatologists and psychologists were first trained and then the efficacy of the patient training program was evaluated based on the causal model of patient education.

**Methods:**

The externally randomized waiting control group study with 249 RA patients included 3 measurement points. The impact of the 5‑h basic training on disease and treatment-related knowledge as well as health competence of RA patients was examined. Secondary questions included attitudinal parameters, communication competence, effects on the disease and satisfaction with the educational program. Data were analyzed on an intention to treat basis by means of covariance analyses for the main target variables, adjusted for baseline values.

**Results:**

The analyses showed that the training program was effective. Even 3 months after training, participants reported more knowledge and health competence than the waiting control group, with small to medium-sized effects (d = 0.37 and 0.38, respectively). With the exception of disease communication, no other effects of training were observed in the secondary objectives.

**Conclusion:**

The basic training program provides a good foundation to develop further interventions to improve attitudinal and disease parameters. It can serve as a central component for rheumatological healthcare for patients with RA at various levels.

Schulungsprogramme sind in der Versorgung von Patienten mit rheumatischen Erkrankungen seit Langem etabliert. Vor dem Hintergrund der neuen Leitlinien und der EULAR-Empfehlungen [21] haben die Deutsche Gesellschaft für Rheumatologie (DGRh), der Verband rheumatologischer Akutkliniken (VRA), die Deutsche Rheuma-Liga (DRL) und die Deutsche Vereinigung Morbus Bechterew (DVMB) begonnen, bisherige Schulungsprogramme zu aktualisieren und neue Angebote zu entwickeln. Diese können aus dem kürzlich publizierten neuen Rahmenkonzept rheumatologischer Schulungsprogramme [23] abgeleitet werden. Für die Patientenversorgung wurde eine 5‑stündige Basisschulung konzipiert und erfolgreich evaluiert. Dabei wurde auf inhaltliche Korrespondenz zur bestehenden strukturierten Patienteninformation StruPI [29] geachtet, um für die Versorgungspraxis thematisch kongruentes Patientenwissen zur Verfügung zu stellen.

## Hintergrund und Fragestellung

Die deutsche Gesellschaft für Rheumatologie erarbeitet seit über 4 Jahrzehnten Schulungsprogramme für Patienten mit rheumatischen Erkrankungen, die von den Betroffenen als bedeutendes Behandlungselement geschätzt werden. Die Wirksamkeit von Schulungen ist grundsätzlich gut belegt [8], es ist aber auch bekannt, dass die Evaluation nicht einfach ist und dass es unterschiedliche Zielgrößen und Messmethoden gibt. Systematische Reviews zeigen, dass die Erkrankung positiv beeinflusst werden kann, indem krankheitsbedingte Einschränkungen vermindert und Schmerzen reduziert werden [5]. Für Patientenschulungsprogramme konnten bisher allerdings nur begrenzte Effekte gezeigt werden [25]. Eine dauerhafte Veränderung von relevantem Gesundheitsverhalten ist für Patienten nicht einfach (z. B. [2]). Daher gibt es auf nationaler und internationaler Ebene Initiativen, etablierte Schulungen unter anderem auf der Grundlage verhaltensorientierter Methoden effektiver zu gestalten [12, 21].

Die Weiterentwicklung von Schulungsprogrammen für Patienten mit rheumatischen Erkrankungen kann durch ein Rahmenkonzept erleichtert werden [23]. Dieses berücksichtigt zum einen den aktuellen Wissensstand in der Rheumatologie und folgt zum anderen international konsentierten Empfehlungen für Psychoedukation [21]. Patientenschulungsprogramme für verschiedene rheumatologische Indikationen wie die häufigste entzündlich rheumatische Erkrankung, die rheumatoide Arthritis (RA), und für unterschiedliche Behandlungseinrichtungen wie stationäre und ambulante Akutversorgung, Rehabilitationszentren und Selbsthilfeorganisationen können flexibel daraus abgeleitet und entwickelt werden. Die Standardisierung durch das Rahmenkonzept gewährleistet, dass unabhängig vom jeweiligen Setting eine vergleichbare hohe Qualität erreicht werden kann. Aus dem Rahmenkonzept abgeleitete Schulungen basieren auf dem übergeordneten Weg und Ziel des Empowerments, was bedeutet, dass Betroffene in die Lage versetzt werden sollen, informierte Entscheidungen in Bezug auf ihre Gesundheit zu treffen, ihre Erkrankung gemeinsam mit ihren Behandlern selbst zu managen und einem gesundheitsförderlichen Lebensstil zu folgen [8].

Für die Versorgung von Patienten mit RA wurde unter Nutzung des Rahmenkonzepts eine 5‑stündige Basisschulung konzipiert, die aus Praktikabilitätsgründen an einem Tag durchgeführt werden kann, aber nicht muss. Die Schulung setzt sich aus lehrzielorientierten Bausteinen zum Krankheitsbild, zur Behandlung und Krankheitsbewältigung zusammen, die aus dem Rahmenkonzept [23] und dem Curriculum RA [24] abgeleitet wurden und sowohl rheumatologische als auch psychologische Elemente und Expertise sinnvoll kombinieren. Im Unterschied zu StruPI [29], die von einem internistischen Rheumatologen und der rheumatologischen Fachassistenz durchgeführt wird, werden die Module der Basisschulung von einem rheumatologischen Facharzt und einem Psychologen angeboten, da die Basisschulung explizit psychologische Module zur Krankheitsbewältigung umfasst. Ziel der vorliegenden Studie war die Überprüfung der Wirksamkeit dieser Basisschulung für Patienten mit RA.

## Methoden

### Studiendesign

Die Evaluation der Basisschulung erfolgte im randomisierten Wartegruppendesign mit 3 Erhebungszeitpunkten vor, unmittelbar nach und 3 Monate nach der Maßnahme. Teilnehmer der Interventionsgruppe (IG) nahmen ohne Wartezeit an der Basisschulung teil. Teilnehmer der Wartekontrollgruppe (WG) absolvierten die Schulung nach Abschluss der Nachbefragung der IG.

Die Evaluationsdaten wurden unmittelbar vor der Schulung (Ausgangsbefragung, T1) sowie 3 Monate danach (Nachbefragung, T3) erhoben. Jeweils am Ende der Schulung (T2) bewerteten alle Patienten die Inhalte und Methoden der Basisschulung.

Die Evaluation der Basisschulung war Teil eines vom Innovationsfond geförderten Forschungsprojekts zu Prozessverbesserungen in der Versorgung von Rheuma-Patienten (StärkeR, Förderkennzeichen: 01NVF17004), unterstützt von der Deutschen Gesellschaft für Rheumatologie (DGRh) und ihrer Ad-hoc-Kommission Patientenschulung.

Die ausgewählten Lehrziele, Methoden und standardisierten Schulungsmaterialien (z. B. Arbeitsblätter, Schaubilder) sind zuvor in einem Manual festgelegt worden [13]. Begleitend wurde ein Train-the-Trainer-Seminar entwickelt, um die beteiligten Dozenten vor Start des Evaluationsprojekts zu schulen.

### Stichprobe

**Figure 1:**
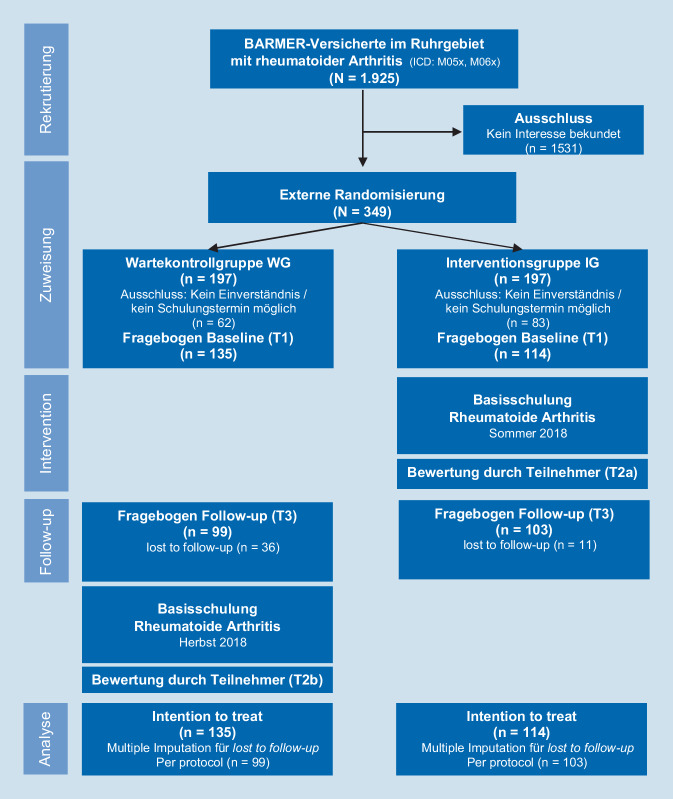

In die Studie wurden erwachsene Patienten aus Nordrhein-Westfalen mit der Diagnose RA (Kodierung M05 oder M06 nach ICD-10) eingeschlossen, die bei der BARMER Krankenkasse versichert waren und die sich in rheumatologischer Behandlung befanden. Von 1925 angeschriebenen und zu einer Teilnahme eingeladenen RA-Patienten bekundeten 394 schriftlich oder telefonisch ihr Interesse an der Basisschulung. Nach einer ausführlichen Information und nach schriftlicher Einverständniserklärung wurden sie in die Studie aufgenommen. Nach entsprechender Dokumentation wurden die Teilnehmer randomisiert und in die beiden Gruppen IG und WG verteilt (Abb. 1). Insgesamt willigten 249 Patienten mit diagnostizierter RA in die Studie ein und beantworteten den Fragebogen T1 (IG: 114, WG: 135). Der Fragebogen T3 wurde von 202 Teilnehmern beantwortet (IG: 103; WG: 99).

Die Schulungsgruppen wurden stets von einem Arzt bzw. einer Ärztin und einem Psychologen bzw. einer Psychologin angeleitet. Alle Schulungsdozenten, die entweder Ärzte mit rheumatologischem Facharztstandard oder mindestens Diplompsychologe waren, hatten zuvor an dem 1‑tägigen Train-the-Trainer-Seminar für die Basisschulung teilgenommen.

### Interventions- und Wartekontrollbedingung

Interventionsbedingung war die Teilnahme an der Basisschulung RA. In der Wartekontrollbedingung erfolgte keine weitere studienbedingte Intervention, d. h. die Teilnehmer wurden weiterhin im Rahmen der Regelversorgung behandelt.

Die durchgeführte Basisschulung bestand aus 4 Inhaltseinheiten von je 50–75 min Dauer, ergänzt durch eine Einführung und eine Pause (Tab. 1). Bei den Modulen Krankheitsbild, Verlauf, Ursachen, Diagnostik und Behandlungsmöglichkeiten wurde darauf geachtet, dass die Informationsmenge auf die Bedürfnisse und die Aufnahmefähigkeit der Teilnehmer abgestimmt war und die Teilnehmer die Inhalte mit eigenen Erfahrungen abgleichen konnten. Durch den Erfahrungsaustausch in der Kleingruppe konnten Faktoren für die Entstehung und Aufrechterhaltung von Schmerzen und eigene Einflussmöglichkeiten besprochen werden. Im Rahmen des 4. Moduls wurden Veränderungen des Lebensstils, Umgang mit Stress und körperlicher Aktivität als Schutzfaktoren besprochen. Die Schulungsinhalte wurden teilnehmerorientiert mithilfe von interaktiven Kurzvorträgen und moderierten Gruppengesprächen vermittelt, um individuelle Informationsbedürfnisse und Patientenerfahrungen themenorientiert in das Schulungsprogramm einzubinden.

**Table 1:** 

Einheiten	Leitung	Dauer
0: Einleitung und Vorstellung	Arzt	15 min
1: Krankheitsbild und Diagnose	Arzt	50 min
2: Behandlung und Krankheitsmanagement	Arzt	60 min
3: Krankheitsakzeptanz, Schmerz- und Alltagsbewältigung	Arzt/Psychologe	75 min
4: Lebensstil, Bewegung und Sport	Psychologe	50 min

Die Basisschulung RA wurde in der IG zunächst 11-mal in Kleingruppen von 5 bis 15 Teilnehmern in 10 rheumatologischen Praxen und Behandlungszentren in Nordrhein-Westfalen durchgeführt. Nach Abschluss der Datenerhebung T3 (Abb. 1) erhielten auch die Teilnehmer der WG die Basisschulung in 11 Kleingruppen.

### Erhebungsinstrumente

Hauptzielgrößen der Evaluation waren das Wissen über die Erkrankung und die Gesundheitskompetenz („health literacy“). Zudem wurden Maße zu Krankheitseinstellungen und -überzeugungen, zur Beschreibung der Erkrankung und des Befindens sowie zur Patientenkommunikation als sekundäre Zielgrößen erhoben.

*Wissenstest und Selbsteinschätzung des eigenen Wissens* Der Wissenstest wurde speziell auf die Inhalte der Schulung abgestimmt. Die 30 Einzelfragen zur RA und ihrer Behandlung konnten mit „Ja – Nein – Weiß nicht“ beantwortet werden (maximale Punktezahl 30). Zusätzlich wurden das selbst eingeschätzte Wissen und die Zufriedenheit mit der Schulung über 6 Items erfasst (Skalenwerte 1 bis 36). Höhere Werte zeigen ein besseres Erkrankungswissen an. Ein analoger Wissenstest und die Skalen zur Selbsteinschätzung des Wissens hatten sich bei der Evaluation anderer rheumatologischer Schulungen bewährt [20].

*Health Literacy Survey (HLS).* Die Gesundheitskompetenz spiegelt das Wissen, die Motivation und die Fähigkeit wider, gesundheitsrelevante Informationen aufnehmen, verstehen und nutzen zu können. Dabei wurden die Bereiche *Informationen verstehen* und *Informationen anwenden* aus dem HLS [26] erfasst (Gesamtindex 1 bis 50). Höhere Werte zeigen eine bessere Gesundheitskompetenz an.

Für die sekundären Zielgrößen wurden folgende Instrumente eingesetzt: Fragebogen zur Krankheitsakzeptanz und -kommunikation [20], Arthritis Self-Efficacy-Scale [19], Rheuma-Kontroll-Skala (RKS) [15], Funktionsfragebogen Hannover (FFbH) [22], Chronic Pain Grade (GCP) [14], RA Disease Activity Index (RADAI) [31], Patient Global Assessment (PGA) [10], Gesundheitsfragebogen PHQ‑4 [17], Kommunikationsbezogene Kompetenz (KOKO) [9] sowie Aktives Informationsverhalten (AIP) [27]. Darüber hinaus wurde der evaluierte Fragebogen zur Schulungszufriedenheit [18] eingesetzt, und es wurden soziodemografische Variablen und Selbstangaben zur Erkrankung und Behandlung erfasst.

### Statistische Auswertung

Die beiden Gruppen IG und WG wurden 3 Monate nach der Schulung (T3) hinsichtlich des Wissens über die Erkrankung und der Gesundheitskompetenz verglichen. Der Gruppenvergleich erfolgte mittels Kovarianzanalyse (ANCOVA) unter Einbezug des jeweiligen Ausgangswertes (T1). Als Effektstärkemaß wurden Cohens d für die adjustierten Mittelwerte berechnet. Werte um 0,20 wurden als kleiner Effekt, um 0,50 als mittlerer und um 0,80 als großer Effekt interpretiert [6]. Die Analyse erfolgte auf Basis von „intention to treat“ (ITT), sodass die Angaben aller Teilnehmer analysiert wurden, auch dann, wenn in der Nacherhebung (T3) kein Fragebogen vorlag. Nicht vorliegende Daten wurden multipel imputiert, wobei auf Skalenebene 10 verschiedene Datensätze erstellt und integriert wurden. Die Auswertung „per protocol“ (PP) für die 202 Teilnehmer, die auch die Fragebögen zur Nachbefragung beantwortet hatten, steht zum Herunterladen zur Verfügung (Tab. S1 und S2). Fehlende Werte einzelner Itemangaben wurden durch multiple Imputation auf Itemebene (10 Datensätze) gehandhabt.^1^ Die Prüfung von Clustereffekten ergab keine relevante Abhängigkeitsstruktur.^2^ Für die Wirksamkeitsanalysen wurden deshalb ANCOVA-Ergebnisse berichtet. Die Analyse für die Nebenzielgrößen erfolgte explorativ. Die Berechnungen erfolgten mit dem Statistikprogramm SPSS, Version 23.

## Ergebnisse

### Teilnehmer

Die durchschnittlich 65 ± 11 Jahre alten Patienten (Altersbereich von 31–87 Jahre) (*n* = 249) waren überwiegend Frauen (84 %). Die mittlere Erkrankungsdauer lag bei 12 ± 11 Jahren (Bereich 1–65 Jahre), die ärztliche Versorgung erfolgte bei fast allen (98 %) durch einen Rheumatologen. Weitere Angaben zu den Teilnehmern finden sich in Tab. 2.

**Table 2:** 

	Wartekontrollgruppe (*n* = 135)		Interventionsgruppe (*n* = 114)		Gesamt (*n* = 249)	
*Lebensalter (Jahre) M (SD)*^a^	65,0	(10,8)	66,06	(11,2)	65,4	(11,0)
*Geschlecht weiblich n (%)*	113	(83,7)	96	(84,2)	209	(83,9)
*Staatsangehörigkeit deutsch n (%)*^b^	131	(100,0)	110	(96,5)	241	(98,4)
*Mit Partner zusammenlebend n (%)*^c^	77	(57,5)	74	(64,9)	151	(60,9)
*Schulabschluss n (%)*^d^						
Volks‑/Hauptschule	57	(42,2)	47	(41,6)	104	(41,9)
Realschule/Polytech. Oberschule	47	(34,8)	46	(40,7)	93	(37,5)
Abitur/Fachhochschulreife	26	(19,3)	17	(15,0)	43	(17,3)
Anderer/keiner	5	(3,7)	3	(2,7)	8	(0,8)
*Berufsausbildung n (%)*^e^						
Lehre	98	(73,1)	67	(58,8)	165	(66,5)
Meister/Fachschule	14	(10,4)	19	(16,7)	33	(13,3)
Universität/Hochschule	13	(6,0)	11	(9,6)	24	(9,7)
Andere/keine	9	(6,7)	17	(14,9)	26	(10,5)
*Erwerbsstatus n (%)*^d^						
Erwerbstätig	41	(30,4)	33	(28,9)	74	(29,7)
Arbeitslos	2	(1,5)	4	(3,5)	6	(2,4)
Rente	85	(63,0)	66	(57,9)	151	(60,6)
Sonstiges	7	(5,2)	11	(9,6)	18	(7,2)
*Berufliche Stellung n (%)*^f^						
Arbeiter	2	(5,0)	3	(9,1)	5	(6,8)
Angestellter	37	(92,5)	29	(87,9)	66	(90,4)
Beamter	0	(0,0)	0	(0,0)	0	(0,0)
Selbstständiger	0	(0,0)	1	(3,0)	1	(1,4)
Sonstiges	1	(2,5)	0	(0,0)	1	(1,4)
*Erkrankungsjahre M (SD)*^g^	11,6	(9,4)	12,4	(10,3)	11,8	(10,8)
*Rh-Faktor n (%)*						
Positiv	74	(57,4)	79	(69,3)	153	(63,0)
(Patient) nicht bekannt	31	(24,0)	24	(21,1)	55	(22,6)
*Behandlung durch Rheumatologe n (%)*	130	(97,7)	111	(98,2)	241	(98,0)
*Mitglied Selbsthilfeorganisation n (%)*	20	(15,2)	26	(23,0)	46	(18,8)
*Schwerbehindertenausweis n (%)*						
Ja	76	(57,1)	75	(65,8)	151	(61,1)
Beantragt	4	(3,0)	1	(0,9)	5	(2,0)

*Anmerkungen.* Fehlende Werte ^a^IG = 1. ^b^WKG = 4. ^c^WKG = 1. ^d^IG = 1. ^e^WKG = 1. ^f^Nur Erwerbstätige (IG: 33; WKG: 41): WkG = 1. ^g^IG = 8, WKG = 13

Die Teilnehmer der beiden Studiengruppen IG und WG waren soziodemografisch sowie in der Ausgangslage der Hauptzielgrößen und Nebenzielgrößen zu Einstellungen und kommunikativen Kompetenzen vergleichbar. Jedoch hatte die WG zu T1 eine etwas geringere Funktionsfähigkeit, mehr Schmerzen, eine höhere Krankheitsaktivität und Globaleinschätzung sowie mehr negatives Befinden als die IG (Tab. 3).

**Table 3:** 

	Ausgangsbefragung (T1)				Nachbefragung (T3)			
	Intervention		Wartekontrolle		Intervention		Wartekontrolle	
	M	(SD)	M	(SD)	M	(SD)	M	(SD)
**Hauptzielgrößen**								
Wissenstest	17,24	(4,95)	18,45	(4,49)	19,84	(5,01)	18,92	(5,82)
Selbsteinschätzung des Wissen und Zufriedenheit	19,76	(6,76)	20,32	(6,41)	23,19	(7,76)	20,44	(9,89)
Gesundheitskompetenz/„health literacy“	32,65	(7,47)	31,38	(7,07)	33,18	(8,16)	29,84	(8,12)
**Nebenzielgrößen**								
*Einstellungen*								
Krankheitsakzeptanz	4,09	(1,13)	3,95	(1,13)	3,99	(1,41)	3,91	(1,32)
Krankheitskommunikation	4,00	(1,25)	3,95	(1,21)	4,24	(1,40)	3,77	(1,50)
Selbstwirksamkeit	6,06	(2,05)	5,68	(1,81)	6,05	(2,33)	5,41	(2,75)
Kontrollüberzeugung	2,76	(0,38)	2,74	(0,38)	2,78	(0,45)	2,78	(0,54)
*Erkrankung und Befinden*								
Funktionsfähigkeit	68,34	(24,64)	65,05	(23,52)	66,12	(24,77)	65,44	(24,52)
Schmerzbelastung	3,91	(2,17)	4,37	(2,05)	3,98	(2,30)	4,09	(2,51)
Krankheitsaktivität	3,70	(1,94)	3,92	(1,83)	3,57	(2,03)	3,69	(2,23)
Globale Selbsteinschätzung der Erkrankung	3,67	(2,33)	3,87	(2,20)	3,49	(2,35)	3,71	(2,75)
Depression und Angst	2,73	(2,35)	3,46	(2,33)	2,88	(2,67)	3,51	(3,02)
*Patientenkompetenz*								
Kommunikationskompetenz	50,97	(26,73)	52,06	(23,81)	50,01	(30,55)	47,03	(29,48)
Aktives Informationsverhalten	2,94	(0,52)	2,85	(0,52)	2,89	(0,64)	2,82	(0,78)

Anmerkungen. M (SD): Mittelwert und Standardabweichung (zur Nachbefragung nicht adjustierte Werte); IG: *n* = 114: WKG: *n* = 135 (10-fach multipel imputierte Datensätze auf Skalenebene für Fälle fehlender Daten zu T3)

### Wirksamkeitsanalysen

Für die Basisschulung zeigen sich positive Effekte auf das Erkrankungswissen und die Gesundheitskompetenz. Die Schulungsteilnehmer der IG erzielten 3 Monate nach der Schulung im Wissenstest einen höheren Punktwert als die WG (Tab. 3). Der Gruppenunterschied unter Einbeziehung der Ausgangswerte (s. Tab. 4) war signifikant (F_1;246_ = 13,30, *p* < 0,001), die Differenz der adjustierten Mittelwerte betrug 1,73 (95 %-Konfidenzintervall [KI]: 0,72–2,75) – ein kleiner bis mittelgroßen Effekt (d = 0,39). Auch die subjektive Einschätzung des Wissens war in der IG höher als in der WG, der Gruppenunterschied unter Einbezug der Ausgangswerte war ebenfalls signifikant (F_1;246_ = 14,45, *p* < 0,001), die Differenz betrug 3,04 (95 %-KI: 1,01–5,07) – ein ebenfalls kleiner bis mittelgroßer Effekt (d = 0,37). Schließlich war auch die Gesundheitskompetenz bei geschulten Teilnehmern höher ausgeprägt als in der WG. Der Gruppenunterschied unter Einbezug der Ausgangswerte war signifikant (F_1;246_ = 11,08, *p* = 0,001). Die Differenz betrug 2,46 (95 %-KI: 0,81–4,11) – ein wiederum kleiner bis mittelgroßer Effekt (d = 0,38). Für die erhobenen Einstellungen, Erkrankungs- und Befindensmaße sowie die kommunikative Patientenkompetenz zeigten sich durch die explorativen Auswertungen der Nebenzielgrößen überwiegend keine weiteren Schulungseffekte. Einzig in der Skala zur Krankheitskommunikation war die IG der WG überlegen – ein kleiner bis mittlerer Effekt (d = 0,36).

**Table 4:** 

	Gruppenvergleich				Effektstärke	
	Differenz	(95 %-KI)	F(1;246)	*p*	Cohen d	(95 %-KI)
**Hauptzielgrößen**						
Wissenstest	1,73	(0,72–2,75)	13,299	<0,001	0,39	(0,14–0,64)
Selbsteinschätzung des Wissen und Zufriedenheit	3,04	(1,01–5,07)	14,452	<0,001	0,37	(0,12–0,62)
Gesundheitskompetenz/„health literacy“	2,46	(0,81–4,11)	11,084	0,001	0,38	(0,13–0,63)
**Nebenzielgrößen**						
*Einstellungen*						
Krankheitsakzeptanz	−0,02	(−0,30–0,25)	0,190	0,663	−0,02	(−0,27–0,23)
Krankheitskommunikation	0,45	(0,17–0,72)	11,312	0,001	0,36	(0,11–0,61)
Selbstwirksamkeit	0,42	(−0,15–1,00)	3,344	0,069	0,18	(−0,07–0,43)
Kontrollüberzeugung	−0,01	(−0,12–0,09)	0,350	0,555	−0,03	(−0,28–0,22)
*Erkrankung und Befinden*						
Funktionsfähigkeit	−2,30	(−5,19–0,59)	3,164	0,077	−0,20	(−0,45–0,05)
Schmerzbelastung	0,26	(−0,17–0,69)	2,165	0,142	0,14	(−0,11–0,39)
Krankheitsaktivität	0,03	(−0,37–0,44)	0,274	0,601	0,02	(−0,23–0,27)
Globale Selbsteinschätzung der Erkrankung	−0,09	(−0,63–0,44)	0,453	0,502	−0,04	(−0,29–0,21)
Depression und Angst	−0,09	(−0,68–0,50)	0,238	0,626	−0,04	(−0,29–0,21)
*Patientenkompetenz*						
Kommunikationskompetenz	3,39	(−3,43–10,21)	1,329	0,250	0,12	(−0,13–0,37)
Aktives Informationsverhalten	0,04	(−0,14–0,22)	0,881	0,349	0,06	(−0,19–0,31)

Anmerkungen. F(df;df) und *p* : ANCOVA-Gruppenvergleich der Nachbefragung (adjustiert für jeweiligen Ausgangswert)

95 %-KI: Konfidenzintervall; IG: *n* = 114; WKG: *n* = 135 (10-fach multipel imputierte Datensätze für Fälle fehlender Daten zu Follow-up)

### Bewertung der Basisschulung aus Teilnehmersicht

Die Basisschulung RA wurde von den Teilnehmern insgesamt sehr gut aufgenommen (Tab. 5). Die überwiegende Zahl der Teilnehmer bewertete die Schulung insgesamt als sehr gut oder gut, nur wenige als befriedigend oder ausreichend. Die Note ungenügend oder mangelhaft wurde gar nicht vergeben. Vergleichbar gut wurde die Schulung auch in den Einzelaspekten der Schulungsinhalte, der interaktiven Gestaltung und der Materialien bewertet, wobei Letztere am kritischsten beurteilt wurden. Fast alle Teilnehmer würden die Schulung weiterempfehlen, die überwiegende Mehrheit sogar ganz sicher.

**Table 5:** 

	*1*	*2*	*3*	4	5	6	M	(SD)	*n*
**Bewertung** ***n*** **(%)**	*Sehr gut*	*Gut*	*Befriedigend*	*Ausreichend*	*Ungenügend*	*Mangelhaft*			
Schulung insgesamt	70	75	17	2	0	0	1,70	(0,70)	164
	(42,7)	(45,7)	(10,4)	(1,2)	(0,0)	(0,0)			
Schulungsinhalte	69	111	18	1		0	1,75	(0,63)	199
	(34,7)	(55,8)	(9,0)	(0,5)	(0,0)	(0,0)			
Interaktive Gestaltung	60	117	19	2	1		1,83	(0,67)	199
	(30,2)	(58,8)	(9,5)	(1,0)	(0,5)	(0,0)			
Materialien	32	109	43	13	1		2,20	(0,81)	198
	(16,2)	(55,1)	(21,7)	(6,6)	(0,5)	(0,0)			
**Empfehlung *n* (%)**	*Ganz sicher*					*Auf keinen Fall*			
Weiterempfehlung	132	50	10	2	0	0	1,39	(0,64)	194
	(68,0)	(25,8)	(5,2)	(1,0)	(0,0)	(0,0)			

Es nutzten 65 % der Teilnehmer auch die Möglichkeit, freie Bewertungen abzugeben (Tab. 6). Die Bemerkungen waren weit überwiegend zustimmend und lobend. Nur in 4 % der Anmerkungen wurden in positivem Tonfall Einschränkungen gemacht, die sich fast ausschließlich auf die zu kurze Dauer der Schulung bezogen. Darüber hinaus wurde der Wunsch geäußert, die Basisschulung auf 2 Einzeltermine für medizinische und psychologische Inhalte aufzuteilen.

**Table 6:** 

**Was hat besonders gut gefallen?**
*Ausgewählte Textbeispiele aus 130 Einzelbeiträgen* „Alle Punkte super“ [383] „Alles zusammen, gute Gruppe zum Austauschen, sehr gute Vortragung“ [265] „Art und Weise des Vortrages. Verständlich, informativ und nicht zu überladen“ [364] „Dass allgemeine Fragen in der Gruppe sehr gut erläutert wurden. Wichtig war für mich das mir meine offenen Fragen gut erklärt wurden“ [025] „Dass man seine eigenen Probleme ansprechen konnte und dazu Lösungsvorschläge erhält“ [285]
**Was kann an der Schulung verbessert werden?**
Mehr Zeit [14-mal], Nicht ganz so lange [13-mal] „Auf einmal war sehr viel; sonst gibt es nichts zu verbessern“ [069] Aufteilen auf 2 (oder mehr) Termine [24-mal] *Kritische Anmerkungen zur Schulung:* Psychologische Informationen zur Krankheitsbewältigung waren zu ausführlich bzw. theoretisch; praktische Empfehlungen kamen zu kurz [3-mal] „Vorstellungsrunde inkl. Krankheitsbild hat sich zum Teil in die Länge gezogen“ [108] „Der Austausch mit anderen Teilnehmern sollte länger sein“ [282*]* *Kritische Anmerkungen zur Durchführung:* Bestuhlung [2-mal] Fragebögen im Rahmen der Studie [3-mal] *Aber auch positive Anmerkungen:* Gut so [10-mal] „Alles gut, weiter so!!“ [332]

Anmerkungen: mit „ “ Aussagenzitat, ohne „ “ inhaltliche Zusammenfassung mit ähnlicher Wortwahl; [ ] anonymisierter Teilnehmercode bzw. Häufigkeit der Nennung

## Diskussion

Unsere Studie zeigt erstmals, dass es möglich ist, auf Basis des flexiblen Rahmenkonzepts für rheumatologische Schulungen [23], des Curriculums und der Materialsammlung RA [24] eine 5‑stündige Basisschulung für die Patientenversorgung zu entwickeln. Zudem wurde ein Train-the-Trainer-Seminar für die Anwender der Basisschulung angeboten, um die durchführenden Ärzte und Psychologen auf die Schulung vorzubereiten. Das neue Schulungsmanual und die dazugehörigen Materialien wurden von Patienten und Dozenten sehr positiv bewertet. Die Durchführbarkeit des 5‑stündigen Schulungskonzepts als Tagesseminar kann als gut machbar und akzeptiert bewertet werden. Einige Patienten wünschten sich eine Aufteilung des medizinischen und psychologischen Teils auf 2 Tage. Dies ist prinzipiell möglich, könnte im Alltag aber an der Machbarkeit scheitern.

Für die Effekte der Basisschulung nach 3 Monaten ergab die Studie klare Hinweise auf eine mittelfristige Wirksamkeit hinsichtlich der Verbesserung von Wissen und von Gesundheitskompetenzen sowie der Krankheitskommunikation im Vergleich zu der noch ungeschulten Wartegruppe. Veränderungen sekundär erfasster einstellungs- und verhaltensbezogener oder medizinischer Parameter zeigten sich dagegen nicht.

Nach dem Wirkmodell der Patientenschulung [8] sind diese Befunde gut erklärbar. Wissen und Gesundheitskompetenz werden von der Schulung unmittelbar angesteuert (proximale Wirkfaktoren). Dagegen unterliegen Einstellungen, Verhalten und medizinische Zielgrößen noch vielfältigen weiteren Einflussfaktoren. Das Wissen über die Erkrankung und über die Behandlungsmöglichkeiten stellt für Einstellungen und Gesundheitsverhalten lediglich *eine* Grundlage dar. Eine Veränderung des Wissens führt aber nicht automatisch zur Veränderung von Einstellungen. Hierfür müssen unterschiedliche Informationsverarbeitungsprozesse stattfinden [3]. Für Veränderungen des Gesundheitsverhaltens wurden verschiedene Bedingungsfaktoren beschrieben, wie z. B. Persönlichkeitsmerkmale, Überzeugungen und Erwartungen, Einstellungen und Normen, Umgebungsfaktoren und Umweltbedingungen [28]. Die Wissensvermittlung im Rahmen einer Patientenschulung ist also nur einer unter mehreren notwendigen Behandlungsbausteinen. Weiterführende Schulungsmodule z. B. zum Erproben alternativer Verhaltensweisen, zum Einüben neuer Fertigkeiten oder zur Handlungsplanung und -selbstkontrolle für die Lebensstiländerung würden zusätzlichen Zeitaufwand erfordern. Mit dem flexiblen Rahmenkonzept für rheumatologische Patientenschulungen liegen entsprechende Schulungsbausteine vor, die prinzipiell ergänzt werden können. Über die Patientenschulung hinaus können aber auch weitere Versorgungsbausteine wie verhaltensbezogene Nachsorge, Selbsthilfe oder Rehabilitationsmaßnahmen mit der Basisschulung verknüpft werden.

Bei der Interpretation der Ergebnisse sind folgende Einschränkungen zu beachten.

1. Die manualgetreue Umsetzung der Basisschulung (Treatmentintegrität) wurde nicht überprüft.

Insgesamt konnte die Basisschulung in der Patientenversorgung erfolgreich umgesetzt werden. Das lag zum einen an der systematischen Ableitung der Basisschulung aus dem Rahmenkonzept und an dem durch das Train-the-Trainer-Seminar standardisierten Vorgehen und zum anderen am Engagement der Schulungsdurchführenden. Insgesamt gab es keine Anhaltspunkte, die manualgetreue Umsetzung der Basisschulung infrage zu stellen – weder durch die episodischen Berichte der Schulungsdozenten noch durch die Teilnehmerevaluation. Eine systematische teilnehmende Beobachtung zur Überprüfung wurde aber nicht durchgeführt.

2. Die Evaluation der Basisschulung erfolgte als kontrollierte Studie mit Wartegruppendesign.

Mit der Mitteilung des früheren bzw. späteren Termins für die Basisschulung waren weder die Teilnehmer noch die Schulungsleiter blind gegenüber der Studienbedingung. In die WG wurden etwas mehr Interessenten eingeschlossen als in die IG. Dies mag ungewöhnlich erscheinen, weil bei Evaluationsstudien üblicherweise die IG die Wunschoption ist. Durch die Zufallszuweisung kann das enttäuschende Gefühl, „nur Kontrollteilnehmer“ zu sein, die Motivation zur Studienteilnahme vermindern. In der vorliegenden Untersuchung war dies, basierend auf den Ergebnissen, aber wahrscheinlich nicht der Fall. Vielmehr könnte die 3‑monatige Wartekontrollphase die Terminplanung im Sinne einer verlängerten Vorlaufzeit vereinfacht haben, auch mit positiver Auswirkung auf die Gesamtteilnehmerzahl. Dafür sprechen auch unsystematische Beobachtungen bei der Studienkoordination.

In der Ausgangslage waren IG und WG hinsichtlich der Haupt- und Nebenzielgrößen vergleichbar, bezüglich der Gesamtbelastung durch die Erkrankung aber nicht. Die WG berichtete zu T1 eine niedrigere Funktionsfähigkeit, eine höhere Schmerzbelastung und eine vermehrte Krankheitsaktivität zusammen mit einer höheren Patientenglobaleinschätzung sowie mehr negatives Allgemeinbefinden. Damit ist es nicht auszuschließen, dass Teilnehmer, die zu T1 etwas schwerer erkrankt waren und zufällig der IG zugewiesen worden waren, deshalb an der Schulung nicht teilnehmen konnten und den zugewiesenen Termin deshalb absagten (Drop-outs). Die WG, die zu T1 schwerer belastet waren, konnten zum 3 Monate späteren Zeitpunkt hingegen möglicherweise teilnehmen, weil sie zu dem Zeitpunkt aufgrund einer Verbesserung der Krankheitsaktivität weniger belastet waren. Unterschiede in der Ausgangslage wurden aber durch die Auswertung mittels Kovarianzanalyse für die jeweilige Zielgröße adjustiert. Die Gesamtergebnisse wurden durch die geschilderten Unterschiede aber nicht kritisch beeinflusst.

Weiterhin haben in der IG weniger Teilnehmer den Fragebogen der Nacherhebung (T3) bearbeitet als in der WG. Die Auswertung „per protocol“ für die 202 Teilnehmer, die die Fragebögen jeweils zu beiden Zeitpunkten beantwortet hatten, ergab vergleichbare Ergebnisse, jedoch mit einer Überschätzung der Effekte.

3. Schließlich ist zu berücksichtigen, dass die Zielgrößen mit Selbstbeurteilungsfragebögen gemessen wurden und damit v. a. die subjektive Perspektive der Teilnehmer widerspiegeln.

Das Erkrankungs- und Behandlungswissen wurde durch einen Wissenstest erfasst, der spezifisch auf die Inhalte der Basisschulung ausgerichtet war, und zusätzlich wurde Wissen indirekt mittels einer subjektiven Globalbewertung gemessen – eine bewährte Vorgehensweise [20]. Für die Erfassung des Wissens über die Erkrankung RA liegt international das Patient Knowledge Questionnaire (PKQ) vor [11], welches 12 Fragen mit 5 Multiple-Choice-Antwortmöglichkeiten umfasst, die sich teilweise jedoch nur geringfügig voneinander unterscheiden, was das Lesen erschweren kann. Da der Fragebogen leider bisher nicht in einer evaluierten deutschen Übersetzung vorliegt, wurde der oben genannte eigene Wissenstest verwendet. In dem verwendeten Wissenstest mit dem Antwortformat Ja – Nein – Weiß nicht waren die Antwortmöglichkeiten vereinfacht. Auf neuere internationale Entwicklungen, die im Rahmen einer auf Grundlage der Item-Response-Theorie kalibrierten Itemdatenbank [7] entwickelt wurden, konnte in dieser Studie noch nicht zurückgegriffen werden. Solche Messmethoden könnten aber zukünftig für die Evaluation von Patientenschulungen genutzt werden.

Unter Gesundheitskompetenz („health literacy“) wird allgemein das Finden, Verstehen, Bewerten und Anwenden von gesundheitsbezogenen Informationen verstanden. Für die Evaluation von Patientenschulungen kann Gesundheitskompetenz als eigener Faktor angesehen werden, der jedoch bisher kaum eingesetzt wurde. Als theoretisches Konstrukt umfasst Gesundheitskompetenz mehr als erkrankungsbezogenes Wissen und wird nicht explizit auf chronische Erkrankungen ausgerichtet gemessen. Gesundheitskompetenz in generischer Form zu erfassen wird aber auch für rheumatische Erkrankungen als wichtig angesehen [4]. In den vorliegenden Ergebnissen hat die IG eine höhere Gesundheitskompetenz erreicht als die WG – dies lässt sich aber nicht auf eine Zunahme der Kompetenzwerte nach der Schulung zurückführen. Warum sich in der WG die Gesundheitskompetenz zu T3 nach den erhobenen Messwerten verringert hat, ist zurzeit noch unklar. Mit den von uns erfassten Aspekten, „gesundheitsbezogene Informationen zu verstehen und anwenden zu können“, wurden nur 2 der 4 Aspekte für Gesundheitskompetenz wichtiger Komponenten abgebildet. So wurde die „Suche und Bewertung von Informationen“ als mögliche eigenständige Fragestellung nicht erfragt. Proximale Zielgrößen im Sinne des Wirkmodells der Patientenschulung sind aus unserer Sicht aber eher „Informationen zu verstehen und anwenden zu können“. Der Zusammenhang von Gesundheitskompetenz mit anderen distalen Zielgrößen und Outcome-Maßen bei rheumatischen Erkrankungen konnte bisher nur vereinzelt aufgezeigt werden, ein einheitliches Bild hat sich bisher nicht ergeben [16]. Insgesamt besteht noch wenig Erfahrung mit dem Konstrukt, obwohl die Auseinandersetzung damit bereits vor Jahren angeregt wurde. Deutschsprachige Instrumente zur Erfassung der Gesundheitskompetenz stehen bisher auch nur sehr begrenzt zur Verfügung, und auch das hier eingesetzte Verfahren war bezogen auf die subjektive Kompetenzzuschreibung und die mangelnde objektive Fähigkeitsmessung schon Gegenstand kritischer Auseinandersetzungen [30]. Die Erfassung als subjektive Kompetenzzuschreibung hat aber ausreichend gute Eigenschaften gezeigt [26]. Eine von uns post hoc durchgeführte Betrachtung zur faktoriellen Struktur des Fragebogens bestätigte ein Bi-Faktor-Modell (d. h. einen Hauptfaktor der Gesundheitskompetenz, auf den alle Items laden, sowie 2 Gruppen/Domän-Faktoren, auf die die jeweiligen Items zusätzlich laden). Für den Hauptfaktor Gesundheitskompetenz haben sich auf Grundlage dieses Modells Hinweise auf Messinvarianz über die Erhebungszeitpunkte ergeben. Damit erscheint ein Response-Shift als Erklärungsansatz für die Ergebnisse [1] unwahrscheinlich. Insgesamt sind weitere Studien zum Konstrukt Gesundheitskompetenz und dessen Erfassung erforderlich.

### Schlussfolgerung

Unsere Studienergebnisse belegen die mittelfristige Wirksamkeit der hier evaluierten Basisschulung. Um darüber hinaus Schulungseffekte zu sichern, sind längerfristige Studien erforderlich, die sicher nicht einfach durchzuführen sind. Nichtsdestoweniger bietet diese Basisschulung eine gute Ausgangsbasis für die Schulung von Patienten mit RA, der weitere Schulungsprogramme für andere Indikationen folgen sollten. Ein Patientenschulungsprogramm für axiale Spondyloarthritis, gefordert durch die DGRh, den VRA und die Deutsche Rheuma-Liga, ist bereits erstellt worden, eine zeitnahe Evaluation ist geplant. Das Ziel, Patienten anzuleiten und zu unterstützen, vorhandene Hilfen im Gesundheitswesen optimal zu nutzen und das eigene Gesundheitsverhalten so anzupassen, dass ein selbstbestimmter Umgang mit der Erkrankung erreicht werden kann, ist für das Gesamtmanagement rheumatischer Erkrankungen essenziell.

## Fazit für die Praxis

Die Basisschulung kann als zentraler Baustein in der Versorgung rheumatologischer Patienten empfohlen werden – nicht nur für die rheumatologische Akutversorgung. Es liegen ein ausgearbeitetes Manual und ansprechende Schulungsmaterialien vor, deren Anwendung in einem Train-the-Trainer-Seminar vermittelt wird. Die Schulung ist effektiv und wird von Patienten und Schulungsdozenten gut bewertet. Um die wirksamen Verbesserungen nachhaltig in der Versorgungspraxis erzielen zu können, müssen jedoch die organisatorischen Voraussetzungen für die Implementierung der Schulungen berücksichtigt werden.

## Caption Electronic Supplementary Material





## Footnotes

1 Ausgenommen waren die Unterfragen des RADAI zur Schmerzangabe in den einzelnen Körpergelenken sowie das Item zur Anzahl der Schmerztage im CPG aufgrund der auffälligen Fehlerraten (7–15 %).

2 Es ergaben sich keine Hinweise auf eine in bedeutsamer Weise abhängige Datenstruktur, nachdem die Schätzung entsprechender Varianzparameter mittels hierarchisch linearer Modelle für lediglich 4 der Zielgrößen numerische Werte lieferte, aus denen sich ICC-Werte von 0,037 (RKS), 0,019 (IAP), 0,009 (subjektive Wissenseinschätzung) und 0,004 (PGA) ergaben.
