# Supplementary material for: Evaluation of a basic educational program for patients with rheumatoid arthritis
Source: Z Rheumatol. 2020 Mar 16;79(8):737–48. [Article in German] doi: 10.1007/s00393-020-00769-4 (PMC8249258; doi:10.1007/s00393-020-00769-4)
Supplement: Supplementary file 1 [file 393_2020_769_MOESM1_ESM.pdf]

Tabelle S1: Zielgrößen in der Analyse „per protocol“

	Ausgangsbefragung (T1)				Nachbefragung (T3)			
	Intervention		Wartekontrolle		Intervention		Wartekontrolle	
	M	(SD)	M	(SD)	M	(SD)	M	(SD)
**Hauptzielgrößen**								
Wissenstest	17,33	(4,82)	18,59	(4,47)	20,02	(4,52)	18,71	(4,61)
Selbsteinschätzung des Wissen und Zufriedenheit	19,88	(6,79)	20,52	(7,12)	23,43	(7,08)	20,15	(6,37)
Gesundheitskompetenz/ Health Literacy	32,88	(7,29)	31,59	(7,25)	33,34	(7,64)	30,10	(7,18)
**Nebenzielgrößen**								
Einstellungen								
Krankheitsakzeptanz	4,06	(1,13)	4,03	(1,11)	3,98	(1,32)	4,02	(1,32)
Krankheitskommunikation	4,02	(1,28)	3,97	(1,31)	4,31	(1,26)	3,69	(1,33)
Selbstwirksamkeit	5,99	(2,10)	5,61	(1,86)	6,01	(2,18)	5,49	(2,34)
Kontrollüberzeugung	2,76	(0,40)	2,75	(0,38)	2,77	(0,39)	2,80	(0,45)
Erkrankung und Befinden								
Funktionsfähigkeit	68,81	(24,06)	66,59	(22,60)	66,60	(23,80)	68,17	(22,50)
Schmerzbelastung	3,85	(2,11)	4,19	(1,99)	3,94	(2,14)	3,84	(2,17)
Krankheitsaktivität	3,69	(1,92)	3,73	(1,76)	3,55	(1,91)	3,53	(1,81)
Globale Selbsteinschätzung der Erkrankung	3,59	(2,28)	3,64	(2,00)	3,46	(2,17)	3,55	(2,32)
Depression und Angst	2,74	(2,36)	3,53	(2,33)	2,86	(2,53)	3,54	(3,97)
Patientenkompetenz								
Kommunikationskompetenz	52,03	(27,66)	52,48	(26,45)	50,38	(26,30)	46,93	(32,70)
Aktives Informationsverhalten	2,95	(0,54)	2,87	(0,55)	2,90	(0,63)	2,81	(0,67)

Anmerkung. M (SD): Mittelwert und Standardabweichung (zu Follow-up nicht adjustierte Werte)
IG: n = 103: WKG: n = 99 (10fach multipel imputierte Datensätze für Fälle fehlender Daten zu Follow-up)
