# Supplementary material for: Evaluation of a basic educational program for patients with rheumatoid arthritis
Source: Z Rheumatol. 2020 Mar 16;79(8):737–48. [Article in German] doi: 10.1007/s00393-020-00769-4 (PMC8249258; doi:10.1007/s00393-020-00769-4)
Supplement: Supplementary file 2 [file 393_2020_769_MOESM2_ESM.pdf]

Tabelle S2: Statistische Analyse „per protocol“

	Gruppenvergleich			Effektstärke	
	mittlere Differenz (Effekt)	(95%-KI)	p	Cohens d	(95%-KI)
**Hauptzielgrößen**					
Wissenstest	2,15	(1,21 - 3,09)	<0,001	0,64	(0,35 - 0,92)
Selbsteinschätzung des Wissen und Zufriedenheit	3,61	(2,01 - 5,22)	<0,001	0,62	(0,34 - 0,91)
Gesundheitskompetenz/ Health Literacy	2,34	(0,80 - 3,88)	0,002	0,43	(0,14 - 0,71)
**Nebenzielgrößen**					
Einstellungen					
Krankheitsakzeptanz	-0,07	(-0,36 - 0,23)	0,575	-0,06	(-0,34 - 0,22)
Krankheitskommunikation	0,59	(0,30 - 0,88)	<0,001	0,57	(0,28 - 0,85)
Selbstwirksamkeit	0,30	(-0,26 - 0,85)	0,227	0,15	(-0,13 - 0,43)
Kontrollüberzeugung	-0,03	(-0,14 - 0,08)	0,474	-0,08	(-0,36 - 0,20)
Erkrankung und Befinden					
Funktionsfähigkeit	-3,55	(-6,34 - -0,76)	0,009	-0,35	(-0,63 - -0,07)
Schmerzbelastung	0,36	(-0,05 - 0,77)	0,069	0,24	(-0,04 - 0,52)
Krankheitsaktivität	0,05	(-0,33 - 0,42)	0,757	0,03	(-0,24 - 0,31)
Globale Selbsteinschätzung der Erkrankung	-0,07	(-0,57 - 0,44)	0,716	-0,04	(-0,31 - 0,24)
Depression und Angst	-0,14	(-0,97 - 0,68)	0,471	-0,05	(-0,33 - 0,23)
Patientenkompetenz					
Kommunikationskompetenz	3,62	(-4,34 - 11,59)	0,267	0,13	(-0,15 - 0,41)
Aktives Informationsverhalten	0,07	(-0,11 - 0,24)	0,319	0,11	(-0,17 - 0,39)

Anmerkung. p: Signifikanz für den ANCOVA-Gruppenvergleich zu Follow-up (adjustiert für jeweiligen Ausgangswert)
95%-KI: Konfidenzintervall
IG: *n* = 103: WKG: *n* = 99 (10fach multipel imputierte Datensätze für Fälle fehlender Daten zu Follow-up)
